# Cerebral infarction 3 weeks after intravenous immunoglobulin for Miller Fisher syndrome: a case report

**DOI:** 10.1186/1752-1947-8-100

**Published:** 2014-03-24

**Authors:** Thashi Chang, Johann Shenoj de Alwis, Neirosha Samarasekara, Senaka Rajapakse

**Affiliations:** 1 Department of Clinical Medicine, Faculty of Medicine, University of Colombo, 25, Kynsey Road, Colombo, Sri Lanka; 2 Durdans Hospital, 3, Alfred Place, Colombo, Sri Lanka

**Keywords:** Cerebral infarct, Intravenous immunoglobulin, Miller Fisher syndrome, Stroke, Thromboembolism, Thrombosis

## Abstract

**Introduction:**

Intravenous immunoglobulin is considered generally safe and is used widely as proven, and sometimes empiric, treatment for an expanding list of autoimmune diseases. Thromboembolic complications following intravenous immunoglobulin therapy are rare and there have been only five previous reports of stroke occurring within 2 to 10 days of infusion. This is the first report of cerebral infarction occurring after a longer latency of 3 weeks following intravenous immunoglobulin therapy in a patient presenting with Miller Fisher syndrome.

**Case presentation:**

A previously well, 44-year-old Sri Lankan man progressively developed ophthalmoplegia, facial paralysis, ataxia and areflexia with neurophysiological and cerebrospinal fluid evidence consistent with the Miller Fisher syndrome. He made an unremarkable recovery with intravenous immunoglobulin therapy (0.4g/kg/day for 5 days, total 180g), but developed a cerebral infarct with haemorrhagic transformation 25 days later. He was noted to have a low blood pressure. Extensive investigations ruled out vasculopathic, embolic, thrombophilic and inflammatory aetiologies. Circulating intravenous immunoglobulins combined with a low blood pressure was considered the most probable cause of his stroke.

**Conclusions:**

Cerebral infarction following intravenous immunoglobulin is thought to be secondary to hyperviscosity, thromboemboli, vasculitis, or cerebral vasospasm and reported to occur after a short latency when the immunoglobulin load is highest. Even though the immunoglobulin load is halved by 3 weeks, our case suggests that that the predisposition to thromboembolism persists over a longer period and may result in vascular complications if synergised with other vascular risk factors. It is recommended that intravenous immunoglobulin be infused at a rate of not less than 8 hours per day and that factors predisposing to thromboembolism such as dehydration, immobilisation and low blood pressure be avoided for the duration of at least two half-lives of immunoglobulin (6 weeks).

## Introduction

Intravenous immunoglobulin (IVIg) is a preparation fractionated from pooled human plasma, to contain primarily immunoglobulin G (IgG). IVIg is increasingly used as an effective treatment for an expanding list of autoimmune diseases. Most adverse effects of IVIg are mild and transient and IVIg is considered generally safe [1]. Thromboembolic complications are recognised but rare, and have been reported to occur in patients with vascular risk factors [2]. There have been only five previous reports of cerebral infarction following IVIg therapy, with reported latencies of 2 to 10 days following infusion [3]. We report the occurrence of cerebral infarction after a longer latency following IVIg therapy for Miller Fisher syndrome (MFS) in a patient with no previous vascular risk factors.

## Case presentation

A previously well, 44-year-old Sri Lankan man presented with perioral and acral paraesthesiae for 3 days associated with disabling, episodic frontal headaches and vomiting. He was afebrile and there was no recent history of fever or symptoms of infection. His general and neurological examinations were normal. His blood counts, inflammatory markers (erythrocyte sedimentation rate, C-reactive protein), renal and liver function tests were normal. A non-contrast-enhanced computed tomography scan of his brain showed no abnormality. Two days after admission to hospital, he developed a right lower motor neurone (LMN) facial paralysis, left partial ptosis and diplopia. His pupils were 3mm bilaterally and reacting to light. Muscle power in his upper and lower limbs was 4+/5 and all deep tendon reflexes were easily elicited. A day later, he developed bilateral LMN facial paralysis, bilateral complete external ophthalmoplegia with bilateral partial ptosis and bilateral dilated pupils with no reaction to light. His muscle power and tendon reflexes remained unchanged, but he was ataxic. His vital lung capacity was 2000mL. Contrast-enhanced magnetic resonance imaging and magnetic resonance angiogram (MRA) of his brain, and electroencephalogram (EEG) were normal. Nerve conduction studies showed focal segmental demyelination with sural sparing. His cerebrospinal fluid (CSF) protein was elevated at 207mg/dL, with no associated cells in the CSF. He was treated with IVIg at 0.4g/kg/day (36g/day) for 5 days. Two days later, he was noted to have global areflexia. He had evidence of syndrome of inappropriate secretion of antidiuretic hormone and required fluid restriction for correction of electrolytes. His blood pressure showed fluctuations from 180/100mmHg to 100/80mmHg and he had a persistent tachycardia. From day 4 of IVIg, he showed improvement in general health, eye movements, facial weakness and incoordination. He was discharged from hospital 11 days after admission. Since he had several high blood pressure readings he was prescribed telmisartan 40mg twice a day.

**Figure 1:**
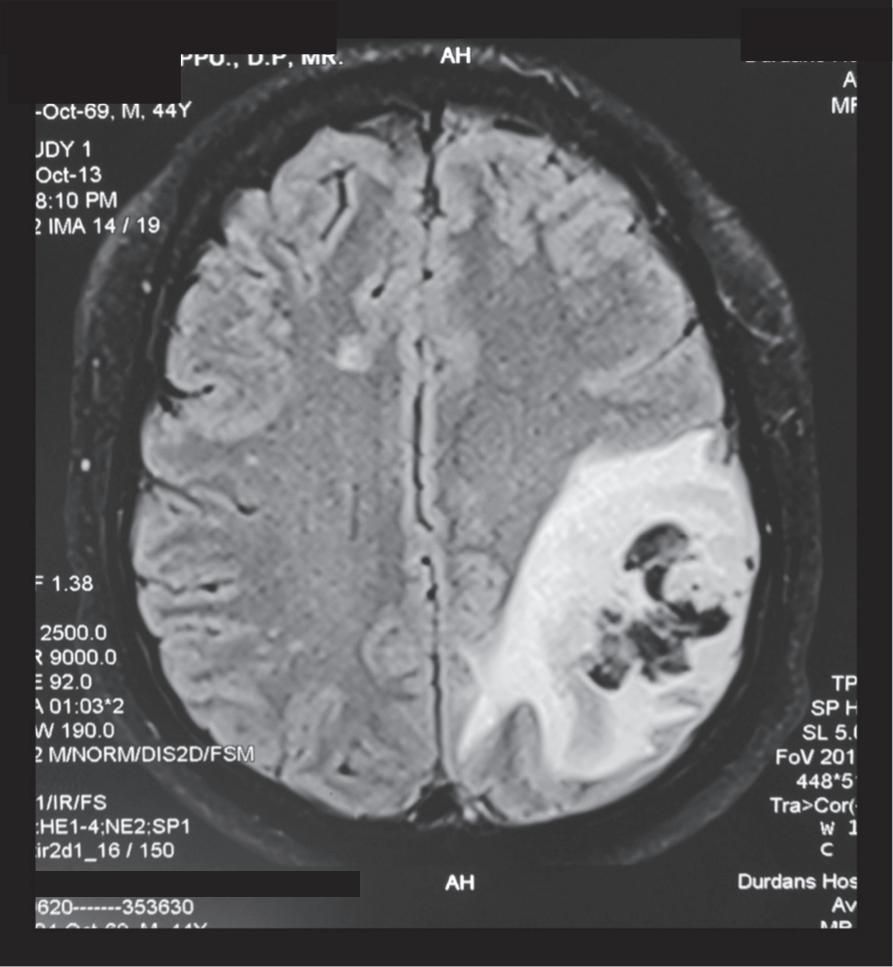
Fluid-attenuated inversion recovery magnetic resonance imaging of brain showing an acute left parieto-occipital infarct with haemorrhagic transformation and perilesional oedema.

On review 3 weeks later, he appeared well with normal eye and facial movements and normal coordination, but complained of persistent headache of 2 days. Optic fundi were normal. His muscle power was almost 5/5 but he had global areflexia. He was noted to have had low blood pressure recordings on home monitoring of 100 to 110/60 to 80mmHg and the telmisartan was reduced to once daily with the proviso of stopping completely after further monitoring. On returning home after review, he had had difficulty in expressing speech and had complained of worsening headache. He was admitted to hospital the next day with recurring secondary generalised seizures and was found to have expressive aphasia and a right homonymous hemianopia. His blood pressure was 100/60mmHg. Brain imaging showed evidence of a left parieto-occipital infarct with haemorrhagic transformation (Figure 1) and the EEG showed left posterior sharp wave discharges. The MRA and venogram were normal. An electrocardiogram, echocardiogram, blood investigations including thrombophilia screening (activated partial thromboplastin time, prothrombin time/international normalised ratio, thrombin time, bleeding time/clotting time and platelet count), plasma glucose and lipid profile, and carotid duplex scan were normal. He was treated with intravenous midazolam, oral sodium valproate and clobazam for seizures; the telmisartan was omitted and intravenous saline was given to restore his blood pressure to 130/80mmHg. He did not have further seizures but complained of increasing headaches, which subsided with 2 days of mannitol and intravenous dexamethasone. He was discharged from hospital 3 days later and had a modified Rankin score of 2 on discharge.

## Discussion

Cerebral infarction following IVIg is rare and thought to be secondary to hyperviscosity, thromboemboli, vasculitis, or cerebral vasospasm [4]. Postmortem neuropathologic analysis of IVIg-associated stroke in Guillain–Barré syndrome (GBS) has shown evidence of intravascular hypercoagulopathy and necrotising microangiopathy but this finding is confounded by the presence of concurrent myeloproliferative disease in the patient [5]. The postulated mechanisms causing IVIg-related stroke are likely to have a short latency since the IVIg load is highest during the immediate post-infusion period. The previous reports of IVIg-related stroke had latencies ranging from 2 to 10 days [3]. Of interest, our patient presented with stroke 25 days following the completion of the IVIg infusion. Given that the half-life of IgG is 23 to 25 days, the IVIg load in our patient at the time of stroke would have been half of the initial loading dose of 180g and the possibility of hyperviscosity, hypercoagulability or vasospasms alone causing stroke seem less likely. However, on a background of low blood pressure combined with half of the loaded IVIg in circulation contributing to the viscosity and the coagulability, it is probable that he had a low-flow cerebral infarct. This is consistent with his stroke occurring in a watershed territory (Figure 1). Our patient had no other vascular risk factors and screening for thrombophilia including antiphospholipid antibodies was negative. A blood pressure of 100/70mmHg by itself is unlikely to have caused a stroke in the absence of vascular risk factors. Furthermore, early haemorrhagic transformation of the infarct suggests thromboembolism rather than pure low-flow as the mechanism of stroke [6].

MFS is characterised by the clinical triad of ophthalmoplegia, ataxia, and areflexia, and is considered a variant form of GBS. In our patient areflexia was slow to develop but subsequently occurred globally. Facial paralysis, although not characteristic, is a recognised feature in 32% to 50% of patients with MFS [7,8]. A preceding infection is not reported in up to a fifth of patients [8]. Clinically, MFS is mostly a self-limiting condition. However, cases progressing to respiratory failure requiring mechanical ventilation and dysautonomia have been described [8]. There are no randomised, double-blind, placebo-controlled trials pertaining to the treatment. The efficacy of plasmapheresis and IVIg has mostly been described in case reports. However, it is reasonable to consider treatment in cases with rapid progression of disease.

## Conclusions

Our case report highlights a rare but severe complication of IVIg therapy and the need for clinical discretion in its use, particularly in self-limiting diseases such as MFS. Furthermore, it is the first report of stroke occurring 3 weeks after IVIg therapy suggesting that predisposition to thromboembolism persists over a longer period and may result in vascular complications if synergised with other vascular risk factors. It is recommended that IVIg be infused at a rate of not less than 8 hours per day and that factors predisposing to thromboembolism such as dehydration, immobilisation and low blood pressure be avoided for the duration of at least two half-lives of immunoglobulin (6 weeks).

## Consent

Written informed consent was obtained from the patient for publication of this case report and accompanying images. A copy of the written consent is available for review by the Editor-in-Chief of this journal.

## Abbreviations

CSF: Cerebrospinal fluid; EEG: Electroencephalogram; GBS: Guillain–Barré syndrome; IgG: Immunoglobulin G; IVIg: Intravenous immunoglobulin; LMN: Lower motor neurone; MFS: Miller Fisher syndrome; MRA: Magnetic resonance angiogram.

## Competing interests

The authors declare that they have no competing interests.

## Authors’ contributions

TC and SR were the primary physicians caring for the patient. JSDA and NS were involved in patient care and contributed academically. TC wrote the manuscript whilst SR critically revised it. All authors read and approved the final manuscript.
